# Total Prosthetic Coverage in Mastopexy with Implants: Total Prosthetic Coverage with Implants Technique

**DOI:** 10.1097/PRS.0000000000012393

**Published:** 2025-08-19

**Authors:** Michele P. Grieco, Santolo D’Antonio, Carolina Arteaga, Jaime Guzmán, Pasquale Tedeschi

**Affiliations:** Rionero in Vulture, Naples, and Bari, Italy; From the 1 Plastic Surgery Unit, IRCCS-Centro di Riferimento Oncologico di Basilicata; 2 Division of Plastic Surgery, AOU Federico II; 3 Department of Precision and Regenerative Medicine of the Ionian Area, Division of Plastic and Reconstructive Surgery, University of Bari Aldo Moro.

## Abstract

**Background:**

Mastopexy with implants is a procedure designed to restore breast shape and volume, often diminished by aging, pregnancy, or weight fluctuations. This study evaluates the aesthetic effectiveness and safety of the total prosthetic coverage in mastopexy with implants (TPC-MI) technique, applied in both retroglandular and dual-plane implant placements. The objective is to ensure proper breast projection and long-term implant stability.

**Methods:**

A prospective cohort study monitored 27 patients who underwent mastopexy with implants from 2021 to 2023, using either the TPC-MI technique (63%) or a dual-plane approach (37%), both combined with dermoglandular flap coverage. The patients, aged 21 to 63 years (mean, 43 years), were evaluated for complications, aesthetic outcomes, and implant stability after surgery. Round silicone implants with different gel cohesivity levels (low, moderate, or high) were selected based on patient-specific tissue characteristics. The aesthetic satisfaction was assessed using the visual analog scale and the Global Aesthetic Improvement Scale.

**Results:**

Postoperative complication rates were low, with a 6% incidence of seroma and a 3% rate of partial areolar necrosis. Aesthetic satisfaction was high, with 80% of patients reporting being “completely satisfied,” according to the visual analog scale, and 92.6% of patients rating their outcomes as “significantly improved” according to th Global Aesthetic Improvement Scale. In addition, the TPC-MI technique demonstrated improved implant stability, significantly reducing the risk of complications such as “bottoming-out.”

**Conclusions:**

The TPC-MI technique is a safe and effective method for breast enhancement, offering high aesthetic satisfaction and low complication rates. The technique’s ability to ensure stable implant placement and natural breast projection makes it a favorable option for patients. Further studies with larger samples and extended follow-ups are recommended to confirm these results and refine the technique.

Mastopexy with implants is a plastic surgery procedure aimed at lifting and restoring breast volume, often compromised by factors such as pregnancy and aging. The choice of implant coverage is crucial, as it can be performed at the muscular or glandular level, each offering specific advantages and disadvantages.^1,2^ Various mastopexy techniques have been described^3^; In our study, we adopted a technique called total prosthetic coverage in mastopexy with implants (TPC-MI), defined as complete implant coverage using a vascularized inferior dermoglandular flap. This approach was applied in both retroglandular and dual-plane implant placements, depending on patient-specific anatomy and tissue characteristics. This study aims to evaluate the aesthetic effectiveness and safety of total implant coverage using a dermoglandular flap, applied in both retroglandular and dual-plane placements to ensure proper projection and long-term stability.

## PATIENTS AND METHODS

This study was conducted between January of 2021 and December of 2023 on a sample of 27 patients. Patient selection adhered to strict inclusion and exclusion criteria, focusing on healthy individuals with realistic expectations regarding outcomes. Patients ranged in age from 21 to 63 years, with a mean age of 43 years.

In our experience, the primary causes of breast ptosis that led patients to undergo mastopexy with implants included several factors. Aging accounted for approximately 30% of cases, as it results in a loss of skin and tissue elasticity. Pregnancy and breastfeeding caused significant changes in breast size and structure, contributing to approximately 25% of cases. Weight fluctuations were another significant factor, responsible for approximately 20% of cases, as they can lead to a loss of tissue elasticity, with an imbalance between adipose and glandular tissue affecting breast shape. Lastly, inadequate support during physical activity contributed to approximately 15% of cases. These findings underscore the importance of accurately evaluating the causes before proceeding with surgery.^4^ Revision rates have been reported at 17% for primary augmentation mastopexy and 23% for secondary cases.^5^

### TPC-MI Technique

**Fig. 1.**
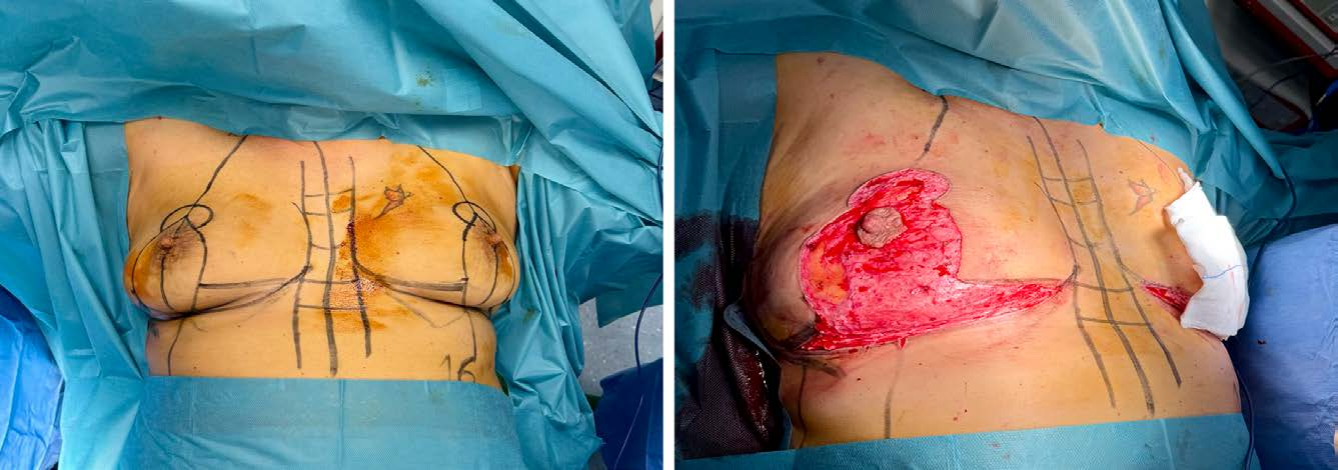
(*Left*) Preoperative design with inverted T scar. (*Right*) Intraoperative deepithelialization of the Wise pattern.

All patients underwent superior pedicle mastopexy using the Wise pattern technique^6^ with an inverted-T scar^7^ (Fig. 1, *left*). This involves an anchor-shaped incision, comprising a perpendicular portion to the nipple and a horizontal section in the inframammary fold (Fig. 1, *right*). (**See Video 1 [online]**, which demonstrates fixation of the inframammary fold with Vicryl 2-0 sutures.) This approach removes excess skin and lifts breast tissue. Operations were performed under general anesthesia, with an average duration of 1.5 hours. The mean sternal notch–to-nipple distance was 25.5 cm, with an average repositioning of the nipple-areola complex to 21 cm.

**Video 1.** This video demonstrates fixation of the inframammary fold with Vicryl 2-0 sutures.

**Fig. 2.**
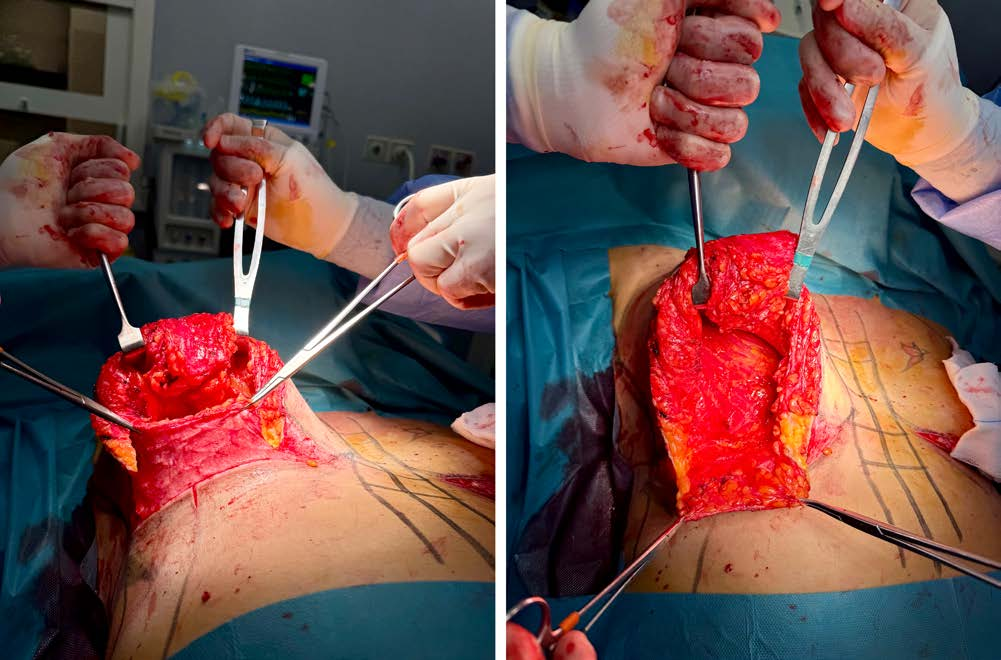
(*Left*) Inferior dermoglandular flap with an average height of 3 cm to 5 cm. (*Right*) Dermoglandular flap and retroglandular pocket.

The TPC-MI technique includes the creation of an inferior dermoglandular flap with an average height of 3 cm to 5 cm (Fig. 2, *left*), which supports the implant and enhances breast aesthetics. Among the patients, 17 (63%) underwent total retroglandular implant placement with complete coverage using an inferior dermoglandular flap (TPC-MI), whereas 10 patients (37%) received a dual-plane implant placement. In both subgroups, the mastopexy technique was identical, and the dermoglandular flap was used to cover the implant and reinforce the inframammary fold (Fig. 2, *right*).

**Fig. 3.**
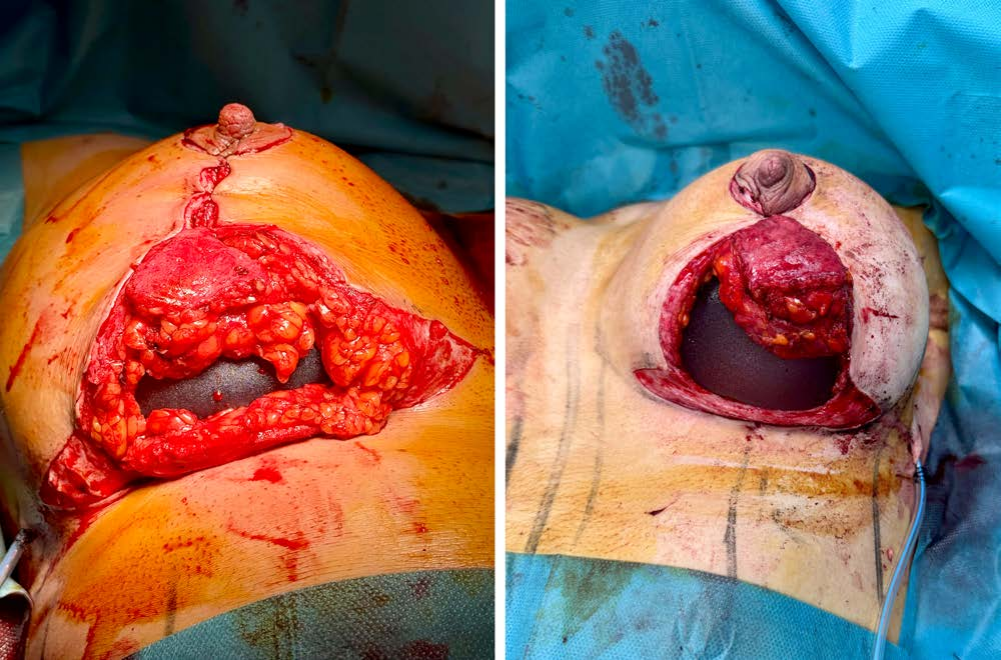
Round retroglandular implant, intraoperative phase. (*Left*) Right breast. (*Right*) Left breast.

All patients received round implants with low (12%), moderate (70%), or high (18%) projection, selected for their ability to deliver more natural and stable long-term results (Fig. 3).

Implant selection was tailored to each patient’s breast tissue characteristics and aesthetic expectations. Three different levels of silicone gel cohesivity were used, as summarized in Table 1.

**Table 1.** Distribution of Implant Cohesivity Levels

Cohesivity Level	No. of Patients	%	Indications
Low cohesivity	4	14.8	Thick subcutaneous tissue, natural softness
Moderate cohesivity	17	63.0	Standard cases with balanced coverage
High cohesivity	6	22.2	Thin tissues, need for projection and support

- Low-cohesive gel implants were used in 4 patients (14.8%), all with adequate subcutaneous fat and glandular coverage, aiming to achieve a softer, natural breast feel.
- Moderate-cohesive gel implants were chosen for 17 patients (63.0%), balancing softness and shape retention, and used in most cases where tissue coverage was average.
- High-cohesive gel implants were used in 6 patients (22.2%), especially in patients with thin tissue envelopes or glandular atrophy, in whom enhanced projection and implant stability were needed.

**Fig. 4.**
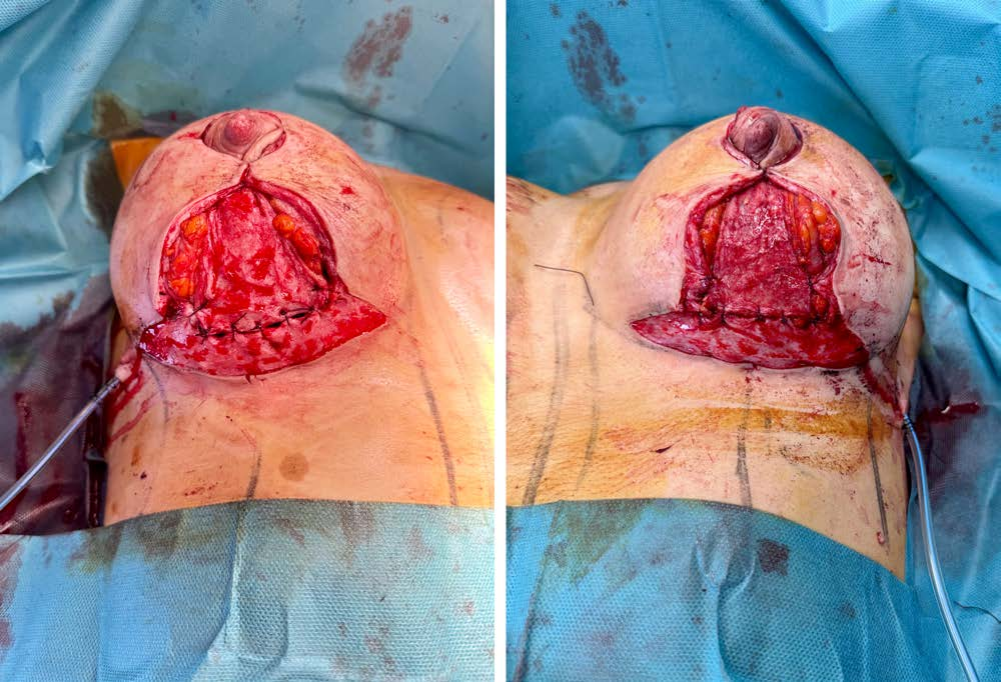
Total glandular coverage in the TPC-MI technique. (*Left*) Right breast. (*Right*) Left breast.

All implants were smooth, round, and ranged in volume from 270 cc to 385 cc, selected according to preoperative dimensional planning and patient preference. Evidence supports the use of round implants for maintaining a more harmonious and durable shape compared with anatomical implants,^8–10^ reducing complications such as rippling and displacement.^11–13^ Total glandular coverage in the TPC-MI technique was achieved by suturing the inferior dermoglandular flap to the superior flap with absorbable Vicryl 2-0 sutures (Fig. 4). Aspirative Hemovac drains were placed in all patients. In addition, the inframammary fold was fixed in all patients using Vicryl 2-0 sutures, with an average of 3 sutures per fold from the prepectoral fascia to the fold.^6^

This fixation reduces the risk of 2 common complications in augmentation mastopexy: “bottoming-out,” which involves implant descent below the inframammary fold; and waterfall deformity, in which the glandular tissue sags over a stable implant. In both the TPC-MI and dual-plane groups, the use of a vascularized dermoglandular flap and fold fixation provided enhanced structural support, preserving projection and breast contour.^10,14–17^ (**See Video 2 [online]**, which demonstrates final stage of placing sutures from the prepectoral fascia to the inframammary fold.)

**Video 2.** This video demonstrates final stage of placing sutures from the prepectoral fascia to the inframammary fold.

Antibiotic prophylaxis was performed intraoperatively with 2 g of cefazolin. All patients continued antibiotic therapy for an additional 6 days, along with systemic analgesics for postoperative pain management and bromelain-based draining medications for 30 days.

## RESULTS

All patients were followed up at 1, 3, 6, and 12 months postoperatively to monitor healing and evaluate aesthetic satisfaction. Statistical analysis revealed a significantly lower complication rate compared with previous studies using dual-plane techniques.^18^ In our experience, 3 patients (11%) developed seroma, which was treated with syringe aspiration, corticosteroid therapy, and lymphatic drainage until complete resolution. One patient (3.7%) experienced areolar necrosis with venous congestion, which resolved with local infiltration of 25,000 IU of sodium heparin and the application of a hyaluronic acid–based ointment. In 1 case (3.7%), liponecrosis of the central part of the inferior dermoglandular flap resulted in implant exposure, requiring subsequent surgical removal of the implant, and revision of the surgical wound. After 4 months, the implant was reinserted using a periareolar approach to avoid further trauma to the previously affected tissue of the inframammary fold (Table 2).

**Table 2.** Postoperative Complications in Patients Using the TPC-MI Technique

Complication	No. of Patients	%
Seroma	3	11
Partial areolar necrosis	1	3.7
Liponecrosis (requiring revision)	1	3.7
Total	5	18.5

**Fig. 5.**
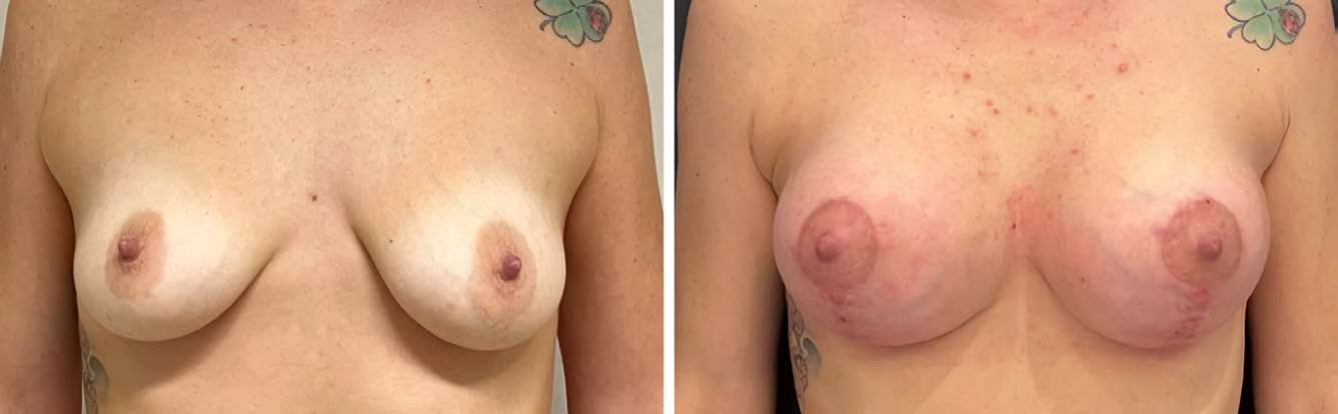
(*Left*) Preoperative and (*right*) 1-year postoperative views.

**Fig. 6.**
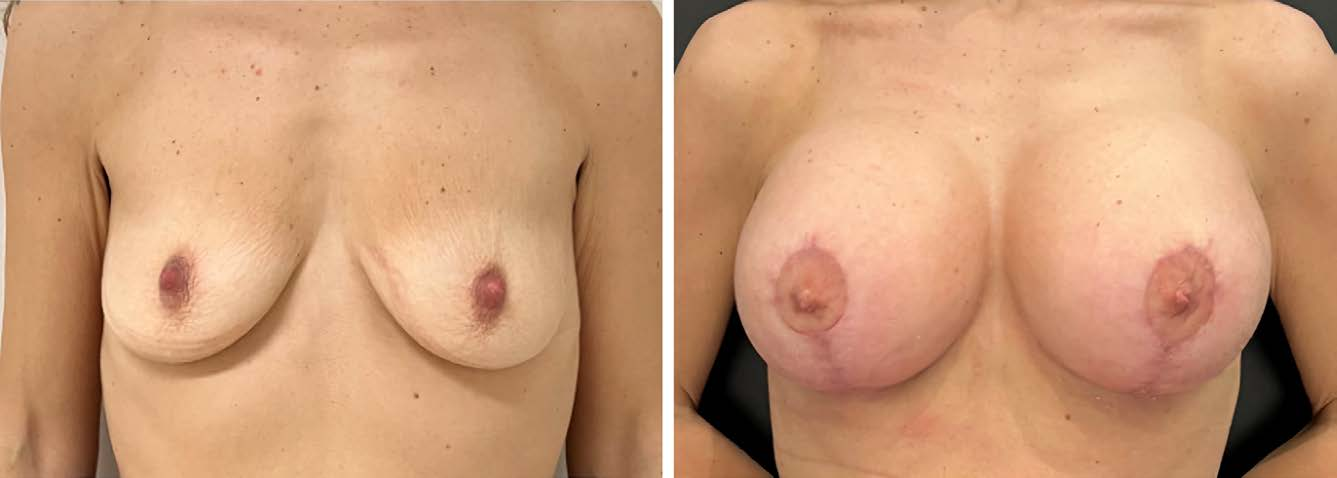
(*Left*) Preoperative and (*right*) 1-year postoperative views.

Patient satisfaction with the results of mastopexy using the TPC-MI technique was evaluated using 2 validated scales: the visual analog scale (VAS)^19^ and the Global Aesthetic Improvement Scale (GAIS).^20^ The VAS, a widely used tool for assessing patient satisfaction regarding various surgical outcomes, particularly aesthetic results, was used in this study. Patients were asked to rate their satisfaction with the shape, size, and overall aesthetic outcome of their breasts on a scale from 0 to 10, where 0 represents “completely dissatisfied” and 10 represents “completely satisfied.” The results showed high levels of satisfaction, with an average score of 8.5 out of 10. Specifically, 22 patients (81.5%) rated their satisfaction as 9 or 10, indicating a high degree of overall satisfaction with the appearance of their breasts after surgery with the TPC-MI technique (Figs. 5 and 6).

In addition to the VAS, the GAIS was used to evaluate overall aesthetic improvement from both the surgeon’s and the patient’s perspectives. This qualitative tool ranges from “significantly improved” to “worsened,” with several intermediate grades. For this study, GAIS assessments were conducted during follow-up visits by both the surgeon and the patient. GAIS results indicated that 92.6% of patients rated their aesthetic outcome as significantly improved, whereas the remaining 7.4% rated it as improved. The high percentage of significantly improved ratings underscores the success of the TPC-MI technique in enhancing breast aesthetics.

These findings are consistent with other studies demonstrating the reliability and validity of the VAS^19^ and GAIS^20^ in evaluating postoperative aesthetic outcomes and patient satisfaction following augmentation mastopexy. Furthermore, these results highlight the importance of addressing the psychological effects of surgery on patients^21^ Table 3.

**Table 3.** Aesthetic Satisfaction Based on the VAS and the GAIS

Satisfaction Scale	No. of Patients	%
VAS (9–10 points)	22	81.5
GAIS (significantly improved)	25	92.6
GAIS (improved)	2	7.4

Regarding implant cohesivity, specific aesthetic advantages were noted in each group. Patients receiving high-cohesive implants (*n* = 6) demonstrated enhanced lower pole projection and shape stability, particularly in those with thin glandular coverage or low body mass index. Conversely, low-cohesive implants (*n* = 4) provided a softer, more natural tactile sensation, suitable for patients with thicker soft-tissue layers. No cases of visible rippling or implant edge palpability were observed in any subgroup, supporting the effectiveness of the TPC-MI dermoglandular flap in achieving full prosthetic coverage, independent of implant cohesivity.

## DISCUSSION

The goal of mastopexy is to restore the shape and volume of the breast by lifting glandular tissue, whereas the use of implants increases breast volume. This study evaluated the aesthetic effectiveness and safety of the TPC-MI technique, which combines superior pedicle mastopexy with round implants and total retroglandular coverage using a dermoglandular flap.

In recent years, both synthetic and biological meshes have been used in aesthetic and reconstructive breast surgery to improve implant support, particularly in patients with poor tissue quality or in revision cases.^22^ Mesh reinforcement, especially in the lower pole, aims to prevent implant malposition, bottoming-out, and fold disruption. However, mesh use introduces additional cost, risk of infection, foreign body response, and potential interference with tissue integration.^23^

The TPC-MI technique offers a valuable alternative by using a vascularized inferior dermoglandular flap, which provides total retroglandular coverage and acts as a biological internal bra. This autologous flap supports the implant and preserves the inframammary fold without introducing any foreign material. By maintaining implant position and enhancing lower pole stability, the dermoglandular flap mimics the mechanical function of mesh while avoiding its complications.^24^

Thus, the TPC-MI technique may effectively eliminate the need for mesh reinforcement, especially in primary procedures or in patients with sufficient glandular volume. This not only enhances biocompatibility and reduces surgical cost, but also aligns with a tissue-sparing philosophy in breast surgery.^25^

The findings confirm that the TPC-MI technique, applied in both retroglandular and dual-plane placements through the use of an inferior dermoglandular flap, offers significant advantages in terms of implant support, projection, and aesthetic reliability. The dermoglandular flap, regardless of implant plane, appears to contribute to lower rates of implant malposition and improved projection.

In retromuscular placement, the long-term risk of implant displacement is frequently encountered.^14,26,27^ However, the TPC-MI technique enhances implant stability, which is crucial for ensuring long-lasting aesthetic results. This technique reduces the risk of implant displacement and rippling, as glandular tissue provides more effective coverage compared with muscle.^18,26,27^

Additional advantages of retroglandular coverage include a more natural breast appearance, as implants placed beneath glandular tissue reduce irregularities and enhance overall breast shape. This technique also allows for a more natural projection of the implant, avoiding the “fake” effect often observed in retromuscular techniques, particularly in the lower pole of the breast. Furthermore, it permits the use of implants of various shapes and sizes, enabling customization based on individual patient preferences.

An important factor influencing surgical outcomes was the cohesivity of the silicone gel used in the implants. In the TPC-MI technique, implant cohesivity was selected based on tissue thickness, breast volume, and desired consistency.

Moderate- to high-cohesion implants ensured long-term shape retention, particularly in patients at higher risk of implant descent or bottoming-out. These implants provided better lower pole support, and maintained upper pole volume and natural projection. In contrast, low-cohesive implants were beneficial in selected cases requiring natural softness, especially when tissue coverage was sufficient.

The ability to tailor cohesivity reinforces the customizability of the TPC-MI technique and emphasizes the value of individualized planning in aesthetic breast surgery. Despite these notable advantages, improper implant placement in retroglandular techniques can lead to complications such as bottoming-out.^10,14–16^ This condition occurs when the implant descends below the inframammary fold, resulting in a flattened and elongated shape, resembling a cascade. Several factors may contribute to this complication, including excess skin tissue, incorrect implant size selection, and biomechanical factors such as implant weight and gravitational force, which can affect implant positioning over time. The TPC-MI technique mitigates this risk through fold fixation, preventing this phenomenon, which negatively impacts aesthetic outcomes, leading to patient dissatisfaction and the need for corrective surgery. As the pectoral muscle is not dissected, patients experience faster recovery and less postoperative pain, with a low incidence of surgical complications, which in our experience is approximately 18.5%.^26^

Another complication to consider is liponecrosis, caused by insufficient blood perfusion. Although rare in our sample, it is significant because the vascular integrity of the inferior mammary pedicle, which supplies blood to the remaining breast tissues, is crucial for surgical success. The impact of overweight and obesity on postoperative complications has been extensively studied in recent decades. Obese patients have been shown to exhibit reduced blood flow in adipose tissue,^28–30^ leading to chronic tissue hypoxia, and subsequent dysfunction and inflammation.^31,32^

Understanding the pathophysiologic mechanisms underlying tissue necrosis and promptly identifying risk factors are essential for reducing morbidity associated with this condition. The sectioning or damage of blood vessels during resection or mobilization of the pedicle reduces tissue perfusion, leading to ischemia and necrosis of adipose tissue. Moreover, liponecrosis can be exacerbated by predisposing factors such as smoking, which has a vasoconstrictive effect and reduces peripheral vascularization, pre-existing vascular abnormalities, obesity, and other factors.^33,34^

The round implants used in this technique not only enhance breast projection but also tend to maintain a more natural appearance and a durable shape compared with anatomical implants.^8,13^ In addition, they help avoid the “deflated” effect typical of anatomical implants.^9,13^ This aspect is particularly relevant for patients seeking harmonious and natural results with a full décolleté. In the long term, our technique demonstrated significant preservation of areolar sensitivity, which is fundamental to quality of life for patients.^35,36^

## CONCLUSIONS

Mastopexy performed using the TPC-MI technique represents a safe and effective option, capable of delivering highly satisfactory aesthetic results, with a reduced incidence of complications. This makes the technique particularly attractive for patients wishing to enhance their appearance without compromising safety. Continued exploration and refinement of mastopexy surgical techniques are essential to ensure optimal care and outcomes for patients. This study highlights the importance of a well-considered and personalized surgical approach, addressing not only physical outcomes but also psychological implications for patients. Further research with larger samples and long-term follow-ups is necessary to confirm these findings and optimize surgical practices in mastopexy.

## DISCLOSURE

The authors declare that they have no conflicts of interest to disclose. This study did not receive any specific grant from funding agencies in the public, commercial, or not-for-profit sectors.

## PATIENT CONSENT

Patients provided written informed consent for the use of their images.

## ACKNOWLEDGMENTS

The authors would like to thank all collaborators and technical staff who contributed to this study.
